# Hookah-Related Posts to Twitter From 2017 to 2018: Thematic Analysis

**DOI:** 10.2196/11669

**Published:** 2018-11-19

**Authors:** Jon-Patrick Allem, Likhit Dharmapuri, Adam M Leventhal, Jennifer B Unger, Tess Boley Cruz

**Affiliations:** 1 Keck School of Medicine of University of Southern California Los Angeles, CA United States; 2 Department of Computer Science University of Southern California Los Angeles, CA United States

**Keywords:** hookah, waterpipe, Twitter, social media, nicotine, flavors, social smoking, infodemiology

## Abstract

**Background:**

Hookah (or tobacco waterpipe) use has recently become prevalent in the United States. The contexts and experiences associated with hookah use are unclear, yet such information is abundant via publicly available hookah users’ social media postings.

**Objective:**

In this study, we utilized Twitter data to characterize Twitter users’ recent experiences with hookah.

**Methods:**

Twitter posts containing the term “hookah” were obtained from April 1, 2017 to 29 March, 2018. Text classifiers were used to identify clusters of topics that tended to co-occur in posts (n=176,706).

**Results:**

The most prevalent topic cluster was Person Tagging (use of @username to tag another Twitter account in a post) at 21.58% (38,137/176,706) followed by Promotional or Social Events (eg, mentions of ladies’ nights, parties, etc) at 20.20% (35,701/176,706) and Appeal or Abuse Liability (eg, craving, enjoying hookah) at 18.12% (32,013/176,706). Additional topics included Hookah Use Behavior (eg, mentions of taking a “hit” of hookah) at 11.67% (20,603/176,706), Polysubstance Use (eg, hookah use along with other substances) at 10.95% (19,353/176,706), Buying or Selling (eg, buy, order, purchase, sell) at 9.37% (16,552/176,706), and Flavors (eg, mint, cinnamon, watermelon) at 1.66% (2927/176,706). The topic Dislike of Hookah (eg, hate, quit, dislike) was rare at 0.59% (1043/176,706).

**Conclusions:**

Social events, appeal or abuse liability, flavors, and polysubstance use were the common contexts and experiences associated with Twitter discussions about hookah in 2017-2018. Considered in concert with traditional data sources about hookah, these results suggest that social events, appeal or abuse liability, flavors, and polysubstance use warrant consideration as targets in future surveillance, policy making, and interventions addressing hookah.

## Introduction

Hookah (or tobacco waterpipe) use has recently grown in popularity in the United States, especially among youth and young adults [1,2]. While exposure to hookah smoke has similar health risks to that of combustible cigarettes [3,4], it is perceived as safer than cigarettes in certain vulnerable groups [5] and is subject to fewer regulations [6]. For example, hookah is offered in many flavors, whereas flavored cigarettes are banned in the United States.

Publicly accessible data from individuals who post information on social media websites (eg, Twitter, Instagram, YouTube) can be efficiently harnessed to quickly capture and describe the context of tobacco use [7-9]. Previous analyses of hookah- related posts to social media websites through the year 2017 provide some information about hookah-related contexts, including the importance and appreciation of stylized waterpipes [10,11], use of hookah in social settings [10], copromotion with alcohol [10], and primarily positive user experiences [12-16]. However, cultural trends, the tobacco product consumer marketplace, and tobacco product health policies are evolving constantly and rapidly. The contexts and experiences associated with hookah use rapidly change as well, making it important to provide up-to-date information on such issues to inform targets for surveillance, policy making, and interventions addressing hookah.

In this study, we demonstrate the utility of collecting data from Twitter to document and describe hookah-related conversations from 2017 to 2018. Our goal was to determine the public’s recent experiences with hookah including understanding the social and environmental contexts in which hookah use occurs. Twitter is used by 24% of US adults (23% of men, 24% of women, 24% of white individuals, 26% of African American individuals, and 20% of Hispanic individuals), with 46% of users on the platform daily [17]. Findings from this study should inform tobacco control policy and prevention efforts and demonstrate the utility in using Twitter data for rapid surveillance of health behaviors and tobacco-related products like hookah.

## Methods

### Data Collection

Twitter posts containing the term “hookah” (or “#hookah”) were obtained from Twitter’s Streaming Application Program Interface (API; the filtered stream using the Twitter4J library for collecting tweets with no gaps in the collection time) from April 1, 2017 to 29 March, 2018. There were a total of 963,954 posts during this time.

### Data Processing

We removed retweets and non-English posts, resulting in 348,834 unique posts that were used for analysis. While the word waterpipe is used in academic papers and presentations to refer to hookah, it is uncommon for individuals to use this term on social media, and it was, therefore, not included in this study [18]. To clean the data, we removed tweets from accounts identified as social bots [19,20] using Botometer (also known as Bot or Not) [21], resulting in a final analytical sample of 176,706 tweets from 90,718 unique users.

The final sample was prepared for analysis, which included the process of basic normalization (eg, remove punctuation, lowercase all text), stop word removal (eg, the words “a” and “the”), normalization of Twitter user mentions (eg, “@janedoe” is converted to “@username”), lemmatization (eg, “cat,” “cats,” “cat’s,” are all converted to “cat”), and nonprintable character removal (eg, emojis) [13]. All analyses relied on public, anonymized data; adhered to the terms and conditions, terms of use, and privacy policies of Twitter; and were performed under Institutional Review Board approval from the authors’ university. To protect privacy, no tweets were reported verbatim in this report.

### Topic Identification Methodology

Initially, we analyzed the tweets using word frequencies (of single words and double-word combinations, also known as one grams and bigrams) and visualized the data through word clouds to identify common topics (Multimedia Appendix 1). From this assessment, the authors came to an expert consensus on several topics including *Person Tagging* (eg, the use of @username to tag another Twitter account in a post), *Buying or Selling* (eg, words indicative of buying, selling, or purchasing hookah), *Appeal* or *Abuse Liability* (eg, words indicative of craving, wanting, needing, enjoying, and loving hookah), *Hookah Use Behavior* (eg, mentions of taking a “hit” of hookah or smoking hookah), *Promotional or Social Events* (eg, mentions of ladies’ nights, parties, etc), *Polysubstance Use* (eg, words indicative of alcohol, marijuana, or other substance use along with hookah), and *Flavors* (eg, use of the words “cinnamon,” “blueberry,” and “watermelon”; Textbox 1). In line with prior research [22,23], we looked for words and phrases that suggested *Dislike of Hookah* (eg, “don’t hookah” and “quit hookah”).

Next, we used Word2Vec, a language modeling technique developed by Google that allows users to learn text representations for creating text classifiers [24]. Word2Vec creates embeddings (eg, numerical representations of words that help capture meaning, semantic relationships, and context) for text by using each word in a corpus to predict the words that usually surround it. In other words, Word2Vec creates word embeddings where semantic relationships between words are preserved. One advantage of this technique is that words that are synonyms will have similar embeddings, whereas words that are antonyms will have dissimilar embeddings. Similarly, in the Word2Vec representations of words, the relationship between “king” and “queen” is equal to the relationship between “man” and “woman.”

We used Word2Vec to find similar words for the one grams and bigrams that we identified per topic in the word cloud stage. This process, along with visual inspection and manual edits, allowed us to expand our word list per topic by identifying words that appeared, in posts, in a similar context as our original keywords. For example, through this process we found that the words “crave,” “love,” “enjoy,” and “need” appeared in posts that were similar to posts that contained the words “want” and “hookah”.

Classification was done by checking for the presence of any one of the keywords (one grams and bigrams) in a tweet. If a tweet consisted of any of the keywords associated with a topic, the tweet was classified as part of that topic. In other words, we used a rule-based classification script written in Python where each tweet was checked for the presence of a specified set of n-grams representing a theme. For each analysis, we present findings in a confusion matrix where the diagonal line indicates the prevalence of a topic and the off-diagonal lines indicate topic overlap. For example, a hypothetical post such as “I’m craving hookah and a beer right now” could be classified under *Appeal or Abuse Liability* and *Polysubstance Use*. The number of posts containing both contents would be found at the intersection of the matrix for these 2 topics or at 2.14% (3824/176,706).

**Themes and common words found in posts along with the word “hookah”; these words are meant to provide further context for each theme, are not exhaustive, and are listed in alphabetical order.**

**Person tagging**

- @username

**Promotional events**

- Bar
- Food
- Friday
- Lounge
- Night
- Party
- Saturday

**Appeal or abuse liability**

- Crave
- Enjoy
- Everyday
- Get
- Like
- Love
- Need
- Want

**Hookah use behavior**

- Hit
- Pass
- Puff
- Smoke
- Used

**Polysubstance use**

- Alcohol
- Beer
- Blunt
- Cigs
- Cocktails
- Drinks
- JUUL
- Liquor
- Margaritas
- Vodka
- Weed
- Wine
- Vape

**Buying or Selling**

- Bought Buy
- Order
- Paying
- Purchase
- Sell

**Flavors**

- Flavors
- Mint
- Cinnamon
- Watermelon
- Blueberry
- Guava
- Grape
- Apple
- Fruit
- Peach
- Orange
- Mango
- Candy

## Results

**Figure 1:**
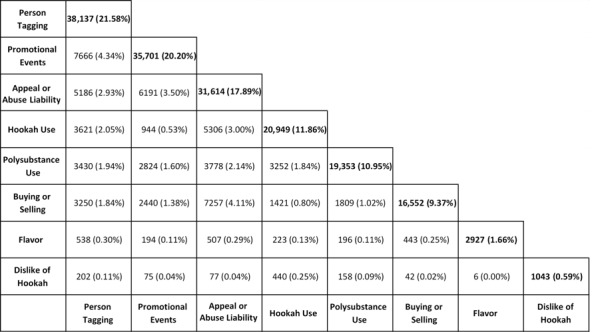
Prevalence of topics.

The total coverage of the 8 topics that we identified constituted 65.45% (115,658/176,706) of all tweets in the corpus of tweets (Figure 1). The remaining 34.59% (61,048/176,706) of tweets were too varied to be classified into a single topic with meaningful coverage (coverage of each subsequent topic was less than 1% of the total tweets). The most prevalent topic was *Person Tagging* at 21.58% (38,137/176,706), followed by *Promotional or Social Events* at 20.20% (35,701/176,706), *Appeal or Abuse Liability* at 18.12% (32,013/176,706), and *Hookah Use Behavior* at 11.67% (20,603/176,706).

About 10.95% (19,353/176,706) of the corpus was *Polysubstance Use*, while *Buying or Selling* comprised 9.37% (16,552/176,706) and *Flavors* comprised 1.66% (2927/176,706) of the tweets. The least common topic was *Dislike of Hookah* at 0.59% (1043/176,706). The most common topic overlap was between *Person Tagging* and *Promotional or Social Events* at 4.34% (7666/176,706), followed by *Buying or Selling* and *Appeal or Abuse Liability* at 4.12% (7276/176,706) and *Promotional or Social Events* and *Appeal or Abuse Liability* at 3.52% (6225/176,706).

## Discussion

### Principal Findings

The topics identified in this study of hookah-related posts to Twitter from 2017 to 2018 provide several insights about the public’s recent experience with hookah. The most prevalent topic was *Person Tagging* or an individual Twitter user directly communicating to another user (a follower or friend) about hookah, while the most common topic overlap was *Person Tagging* and *Promotional or Social Events.* These findings demonstrate that Twitter users communicate shared values around, and experiences with, hookah. In other words, such posts may notify others about hookah-related events and align people into a community around hookah. Similarly, recent research characterizing JUUL-related posts to Twitter found instances of *Person Tagging* where posts suggested that people were notifying their friends of when they were using or purchasing JUUL-related products [22]. Collectively, these interpersonal communications suggest that people bond around tobacco-related products on Twitter and that there may be co-use of tobacco among many people or social influences in which one person motivates another to use tobacco.

*Hookah Use Behavior* and *Polysubstance Use* were identified as topics of discussion and may represent a syndrome of risky behavior among select Twitter users. These findings are in line with earlier research on hookah posts to Tumblr [18] and Instagram [10] as well as survey-based research that demonstrated that those who use hookah were significantly more likely to use other substances including alcohol, cigarettes, marijuana, and cocaine compared with those who refrained from hookah use [25]. Individuals who combine the use of hookah with other substances may be at risk for substance misuse; for example, hookah use facilitates greater intake of alcohol and vice versa [26].

Posts in this study reflected Twitter users’ interest in flavors, which is similar to earlier research on tobacco-related post to Twitter [22,27]. A recent study identified that flavors were a common reason for hookah use among a nationally representative sample of young adults (aged 18-24 years) [28]. Research has also documented that flavored tobacco products like hookah are perceived to be less harmful than cigarettes [29]. Restricting flavors, such as those identified in this study (Cinnamon, Watermelon, Blueberry, etc), to reduce the appeal of hookah may be a policy consideration to explore in the future.

Many posts found in this study reflected that Twitter users craved, enjoyed, or wanted hookah; this finding, when coupled with the finding that posts indicative of disliking hookah were rare, suggests that there is a current need for targeted interventions to discourage the appeal of hookah use. The common discussions about hookah’s appeal may help normalize hookah use on Twitter, which may have consequences for offline behaviors [30].

### Limitations

This study focused on posts to Twitter, and findings may not generalize to other social media platforms. The posts analyzed in this study were collected from a 12-month period and may not generalize to other time periods. While only one root word “hookah” (or “#hookah”) was used in data collection, research has indicated that this is the common term to refer to waterpipe use on social media [10,13,18]. Data collection relied on Twitter’s Streaming API, which prevented collection of tweets from private Twitter accounts. As a result, findings may not represent the attitudes and behaviors of individuals with private accounts.

### Conclusion

Social events, appeal or abuse liability, flavors, and polysubstance use were common contexts and experiences associated with Twitter discussions about hookah in 2017-2018. Considered in concert with traditional data sources about hookah, these results suggest that social events, appeal or abuse liability, flavors, and polysubstance use warrant consideration as targets in future surveillance, public policy, and interventions addressing hookah. This study also highlights a clear benefit of using social media data in public health surveillance. Data from social media can serve as an ongoing system to inform public health researchers about tobacco products or ways in which these products are used by the public in near real time.

## Appendix

Multimedia Appendix 1 Hookah word clouds.

## Abbreviations

API: application program interface
