# Supplementary material for: Hookah-Related Posts to Twitter From 2017 to 2018: Thematic Analysis
Source: J Med Internet Res. 2018 Nov 19;20(11):e11669. doi: 10.2196/11669 (PMC6277830; doi:10.2196/11669)
Supplement: Multimedia Appendix 1 [file jmir_v20i11e11669_app1.pdf]

Hookah Word Clouds (April 2017 – March 2018)

April 2017

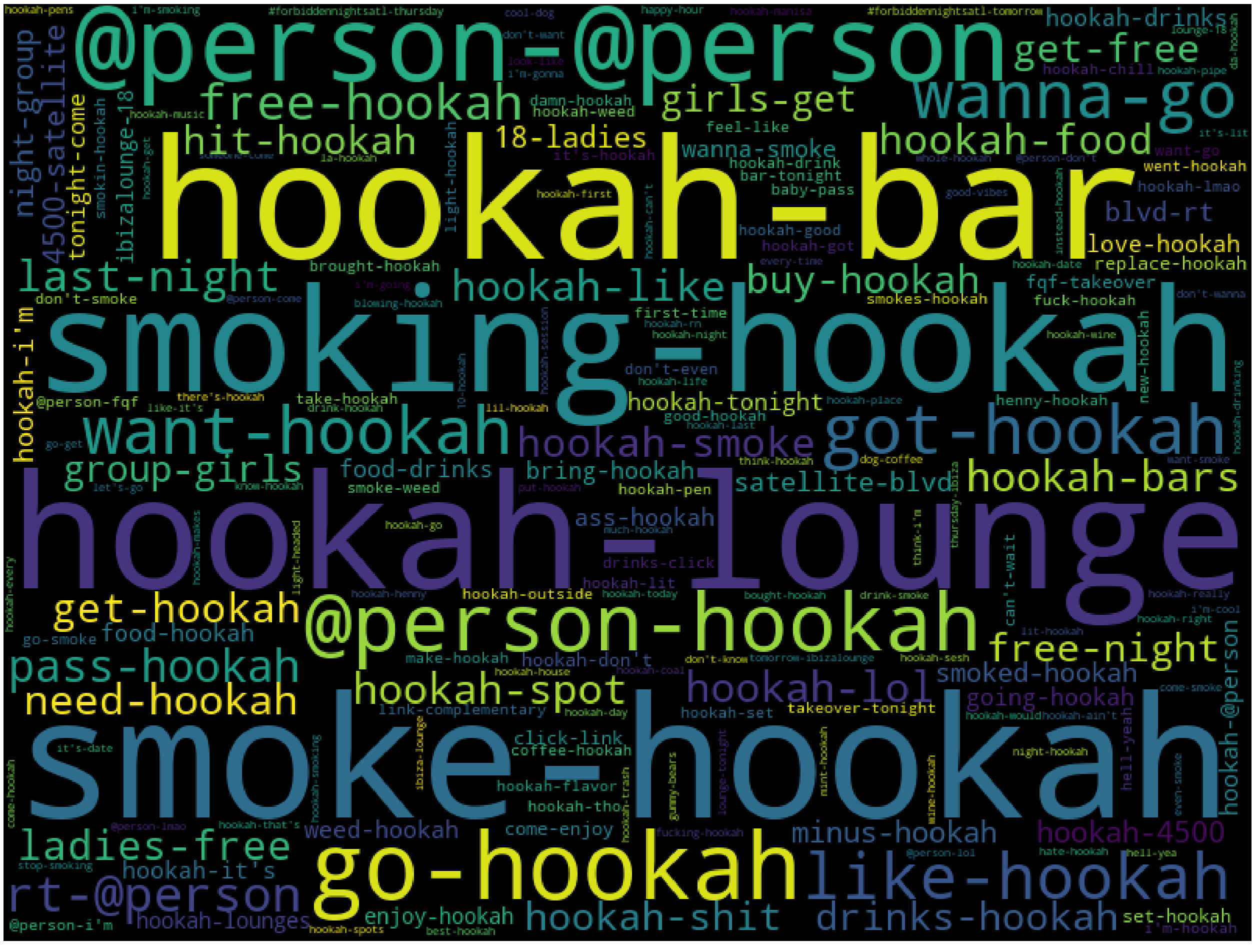

May 2017

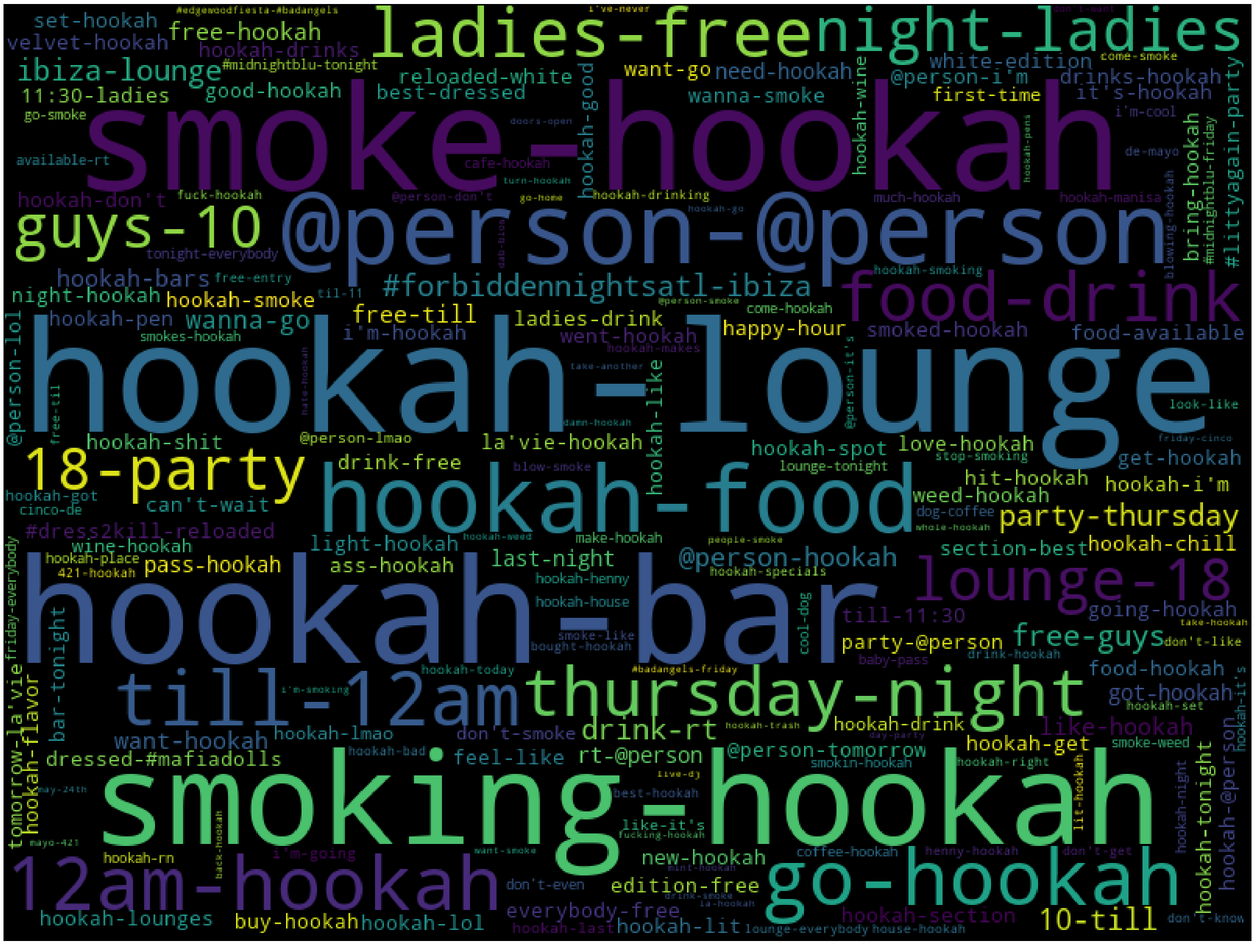
smoke-hookah
hookah-lounge
hookah-bar
smoking-hookah
@person-@person
hookah-food
ladies-free
night-ladies
food-drink
till-12am
thursday-night
12am-hookah
go-hookah
lounge-18
18-party
guys-10
set-hookah
velvet-hookah
free-hookah
hookah-drinks
ibiza-lounge
11:30-ladies
good-hookah
reloaded-white
best-dressed
want-go
need-hookah
wanna-smoke
white-edition
@person-i'm
drinks-hookah
it's-hookah
first-time
hookah-good
hookah-wine
bring-hookah
#littyagain-party
hookah-don't
fuck-hookah
hookah-drinking
much-hookah
hookah-manisa
hookah-bars
night-hookah
hookah-smoke
#forbiddennightsatl-ibiza
hookah-pen
wanna-go
free-till
ladies-drink
i'm-hookah
went-hookah
happy-hour
smoked-hookah
food-available
@person-lol
hookah-shit
@person-lmao
la'vie-hookah
hookah-like
hookah-spot
love-hookah
get-hookah
drink-free
lounge-tonight
hit-hookah
hookah-i'm
weed-hookah
party-thursday
hookah-got
can't-wait
#dress2kill-reloaded
wine-hookah
light-hookah
last-night
@person-hookah
section-best
hookah-chill
hookah-place
pass-hookah
ass-hookah
hookah-henny
hookah-house
going-hookah
till-11:30
party-@person
free-guys
bar-tonight
friday-everybody
tomorrow-la'vie
hookah-flavor
drink-rt
hookah-drink
food-hookah
got-hookah
like-hookah
want-hookah
hookah-lmao
don't-smoke
feel-like
rt-@person
@person-tomorrow
hookah-get
dressed-#mafiadolls
hookah-tonight
hookah-@person
new-hookah
coffee-hookah
edition-free
hookah-rn
i'm-going
don't-even
everybody-free
hookah-section
10-till
hookah-lounges
buy-hookah
hookah-lol
hookah-lit
hookah-last
lounge-everybody
house-hookah

June 2017

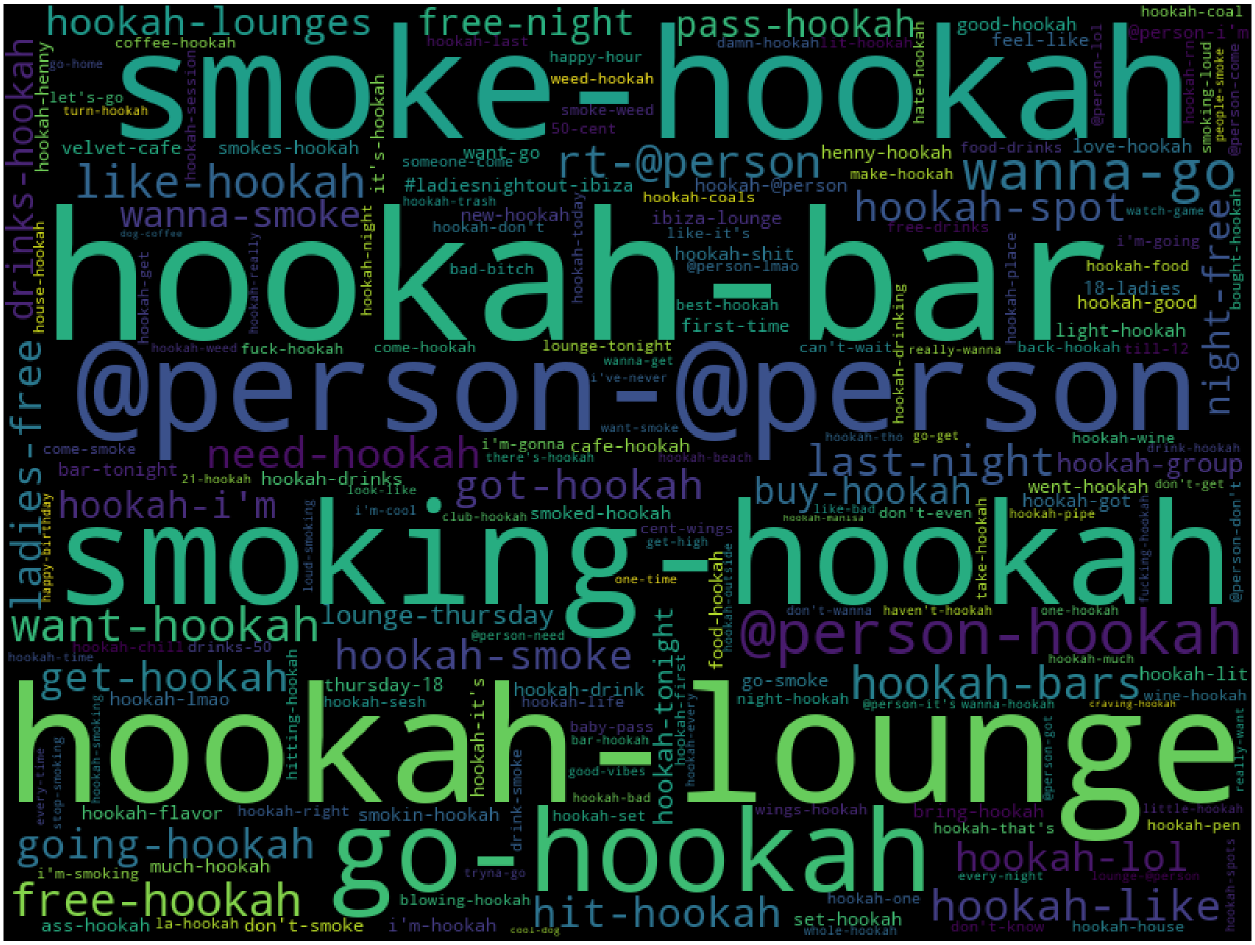
hookah-lounge
hookah-bar
smoke-hookah
smoking-hookah
@person-@person
go-hookah
@person-hookah
hookah-lounges
free-night
pass-hookah
rt-@person
wanna-go
like-hookah
hookah-spot
wanna-smoke
need-hookah
got-hookah
last-night
hookah-group
buy-hookah
hookah-i'm
want-hookah
hookah-smoke
get-hookah
hookah-bars
going-hookah
hookah-lol
free-hookah
hit-hookah
hookah-like
ladies-free
drinks-hookah
night-free
hookah-tonight
lounge-thursday

July 2017

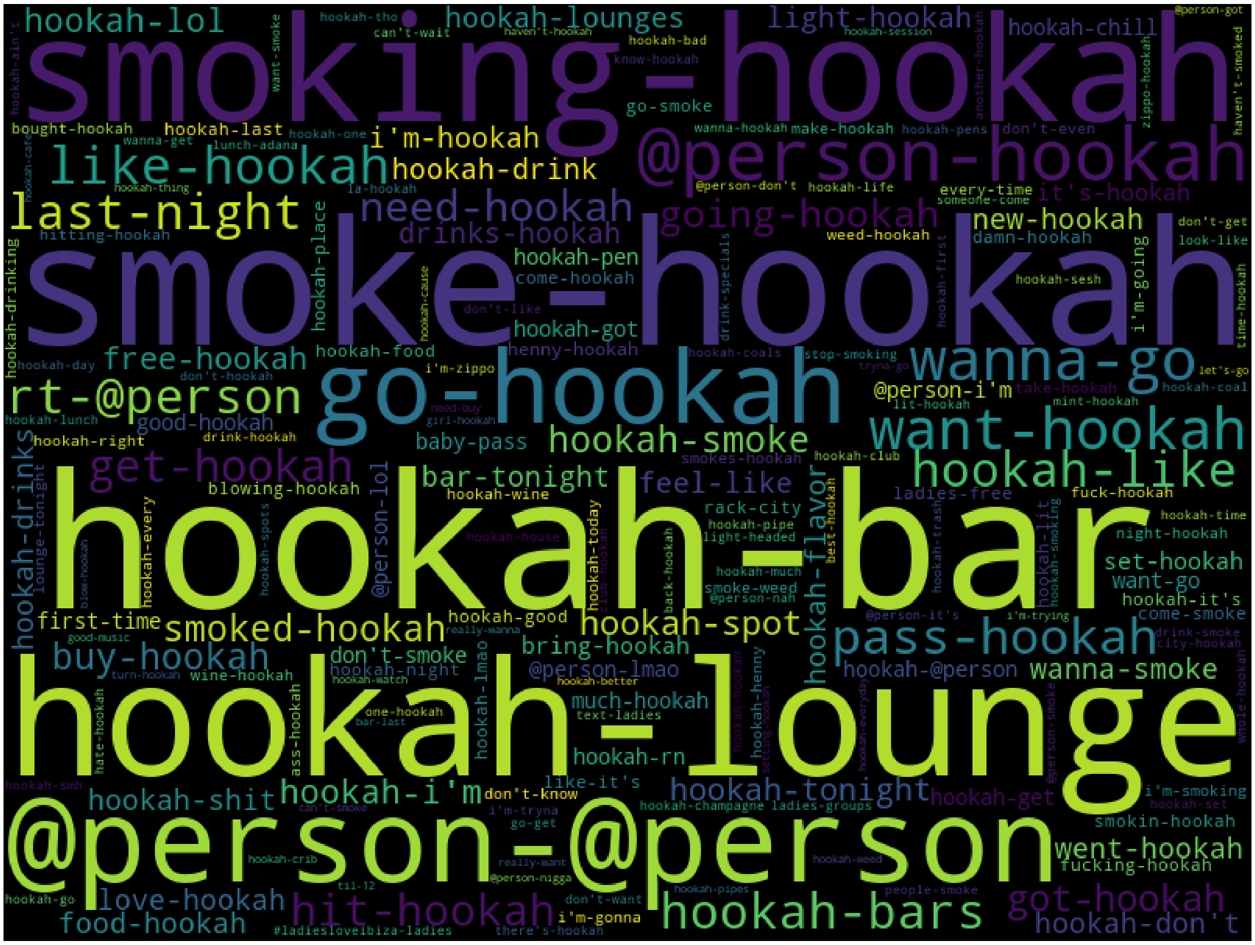
smoking-hookah
hookah-lol
hookah-lounges
light-hookah
hookah-chill
@person-hookah
like-hookah
hookah-drink
i'm-hookah
last-night
need-hookah
going-hookah
new-hookah
drinks-hookah
smoke-hookah
hookah-pen
hookah-got
free-hookah
go-hookah
wanna-go
rt-@person
want-hookah
hookah-smoke
get-hookah
hookah-like
bar-tonight
feel-like
hookah-bar
set-hookah
want-go
hookah-drinks
hookah-flavor
first-time
smoked-hookah
hookah-spot
pass-hookah
buy-hookah
bring-hookah
don't-smoke
hookah-lounge
wanna-smoke
much-hookah
hookah-rn
hookah-shit
hookah-i'm
hookah-tonight
hookah-get
@person-@person
went-hookah
love-hookah
hit-hookah
hookah-bars
got-hookah
food-hookah
hookah-don't

August 2017

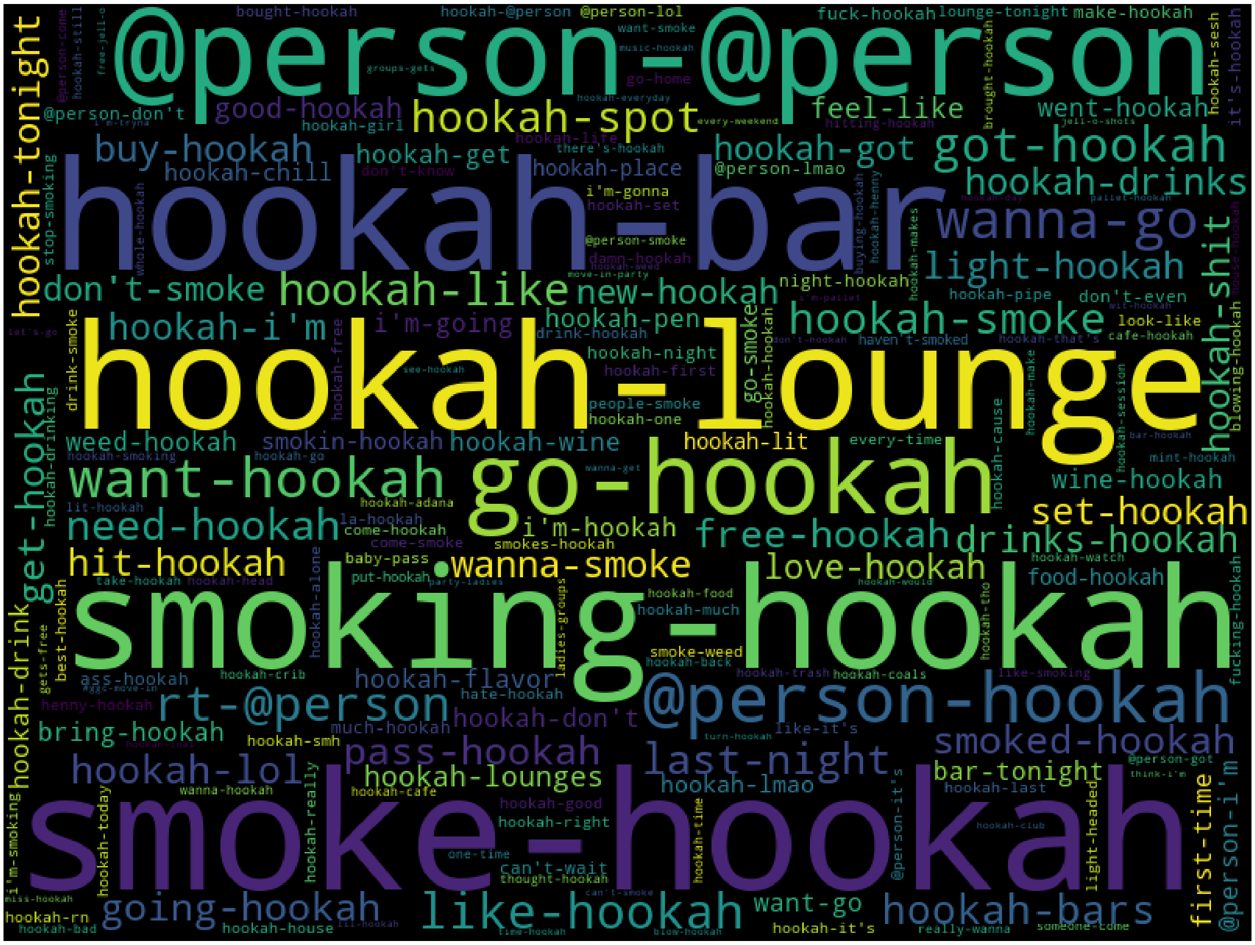

@person-@person
hookah-bar
hookah-lounge
smoking-hookah
smoke-hookah
go-hookah
want-hookah
need-hookah
hit-hookah
@person-hookah
rt-@person
last-night
pass-hookah
hookah-lol
like-hookah
going-hookah
hookah-bars
hookah-tonight
get-hookah
hookah-drink
hookah-spot
buy-hookah
good-hookah
hookah-get
hookah-got
got-hookah
hookah-drinks
wanna-go
light-hookah
hookah-shit
hookah-like
don't-smoke
new-hookah
hookah-smoke
hookah-i'm
hookah-pen
weed-hookah
smokin-hookah
hookah-wine
hookah-lit
wine-hookah
set-hookah
free-hookah
drinks-hookah
i'm-hookah
wanna-smoke
love-hookah
food-hookah
hookah-flavor
smoked-hookah
hookah-lounges
bar-tonight
bring-hookah
hookah-lmao
first-time
@person-i'm
want-go
feel-like
went-hookah
hookah-chill
hookah-place
i'm-going
fuck-hookah
lounge-tonight
make-hookah
bought-hookah
hookah-@person
@person-lol

September 2017

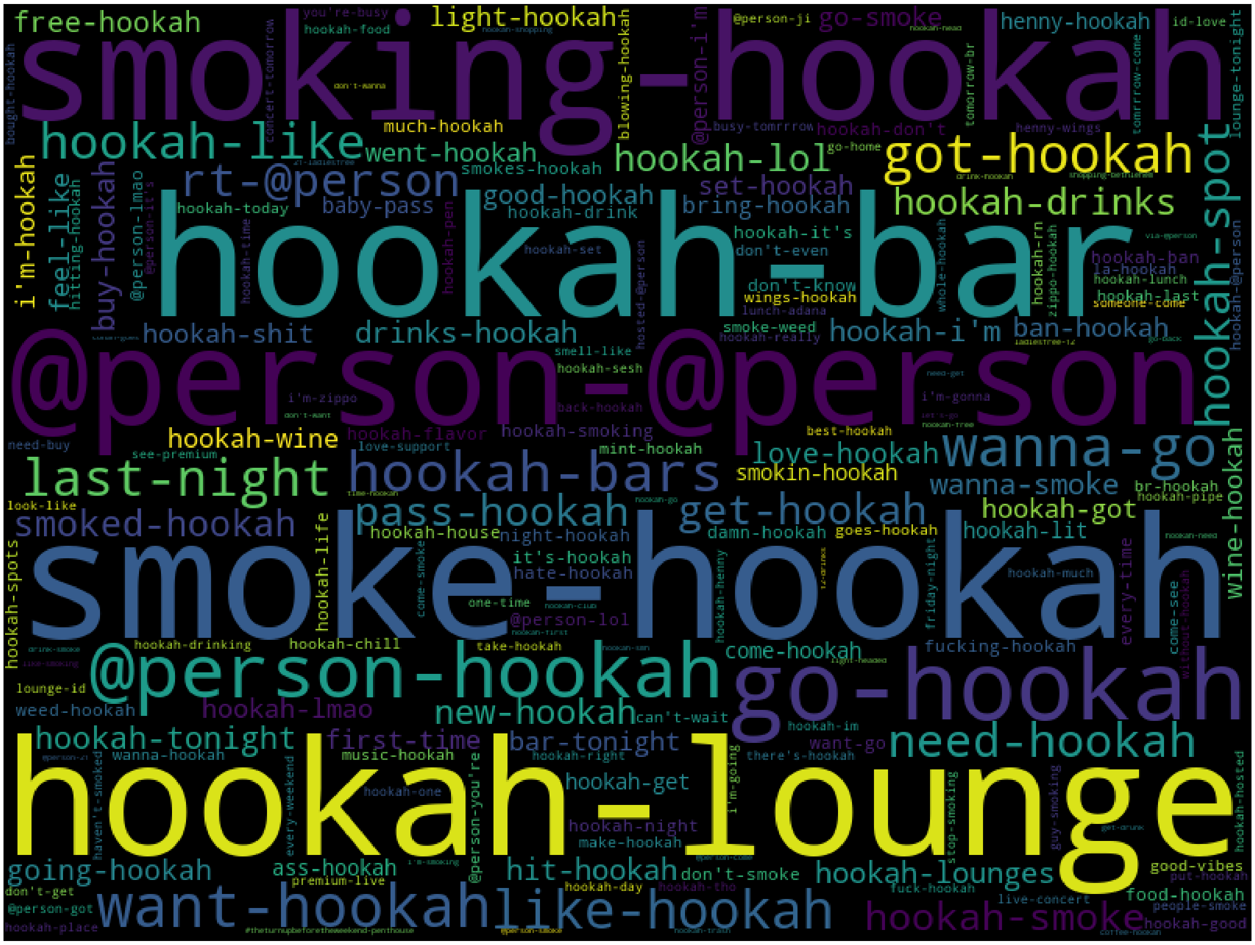

October 2017

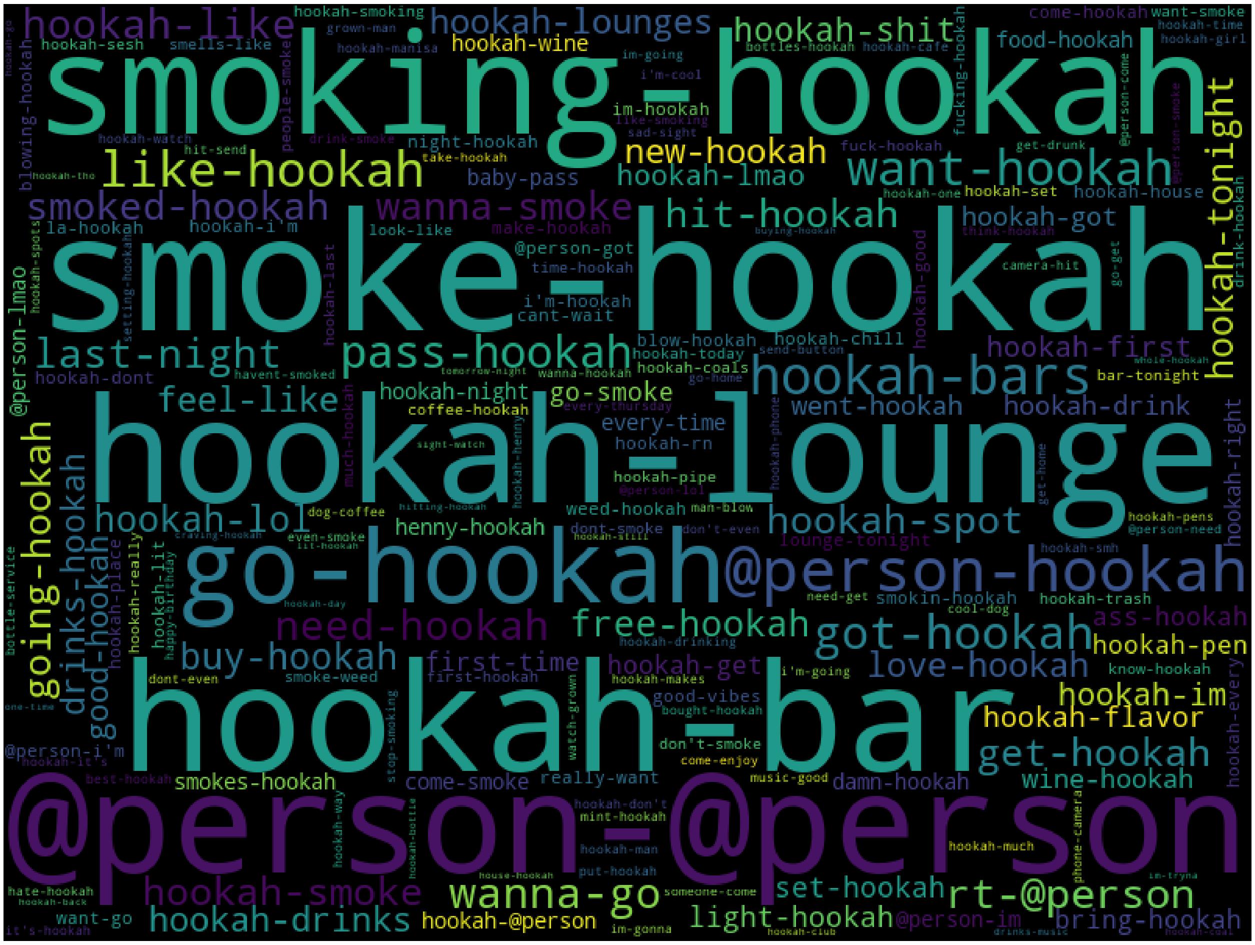

November 2017

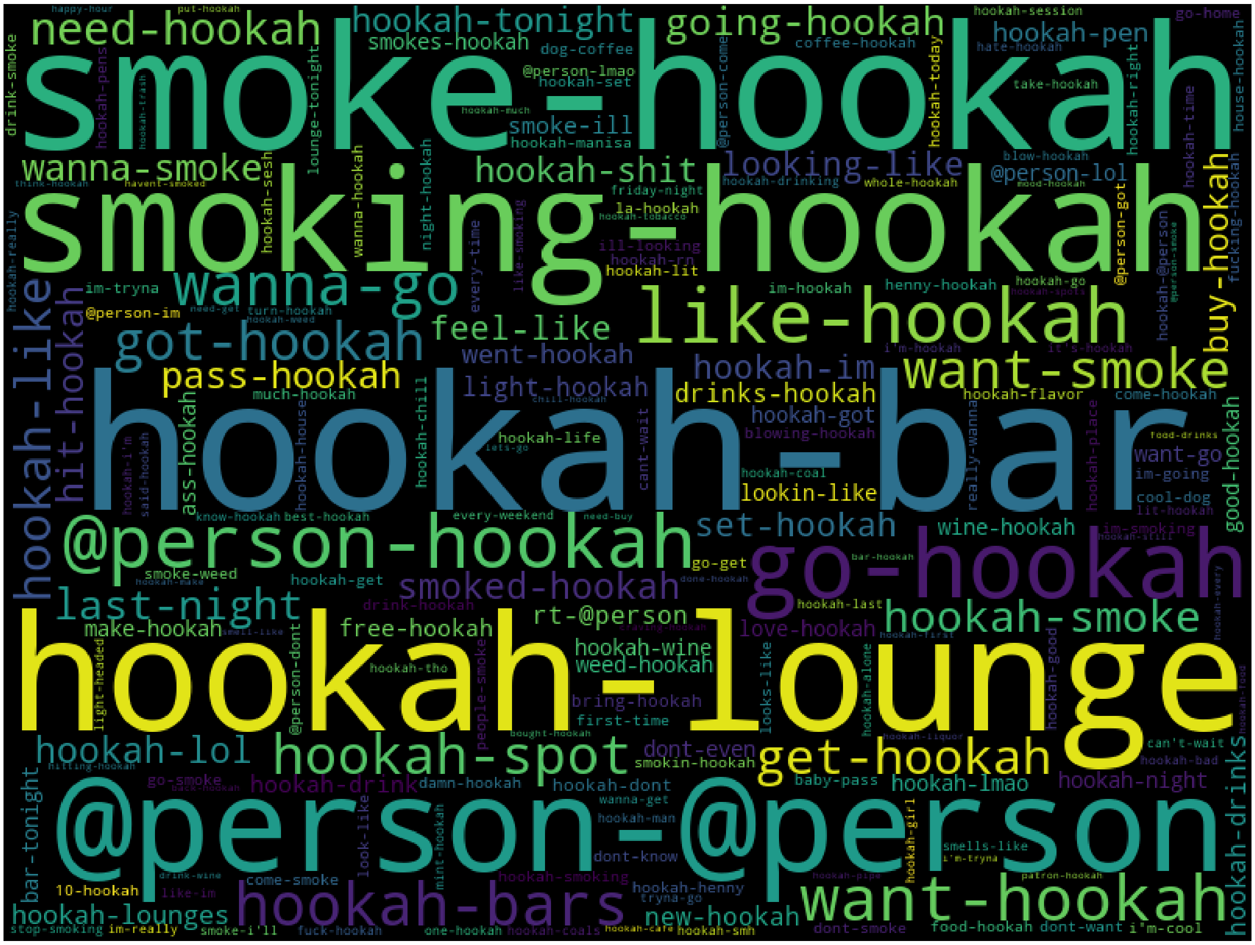

December 2017

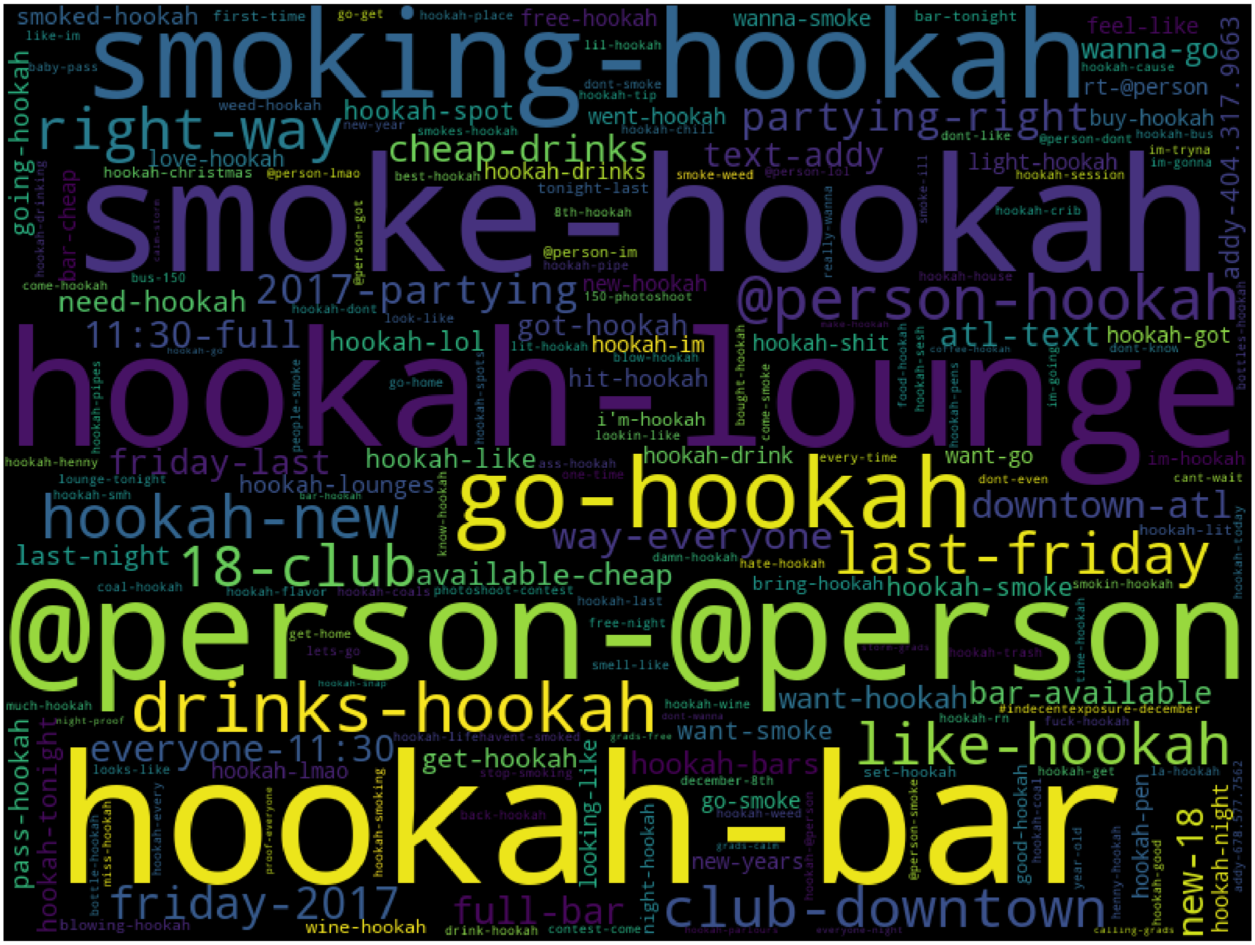

January 2018

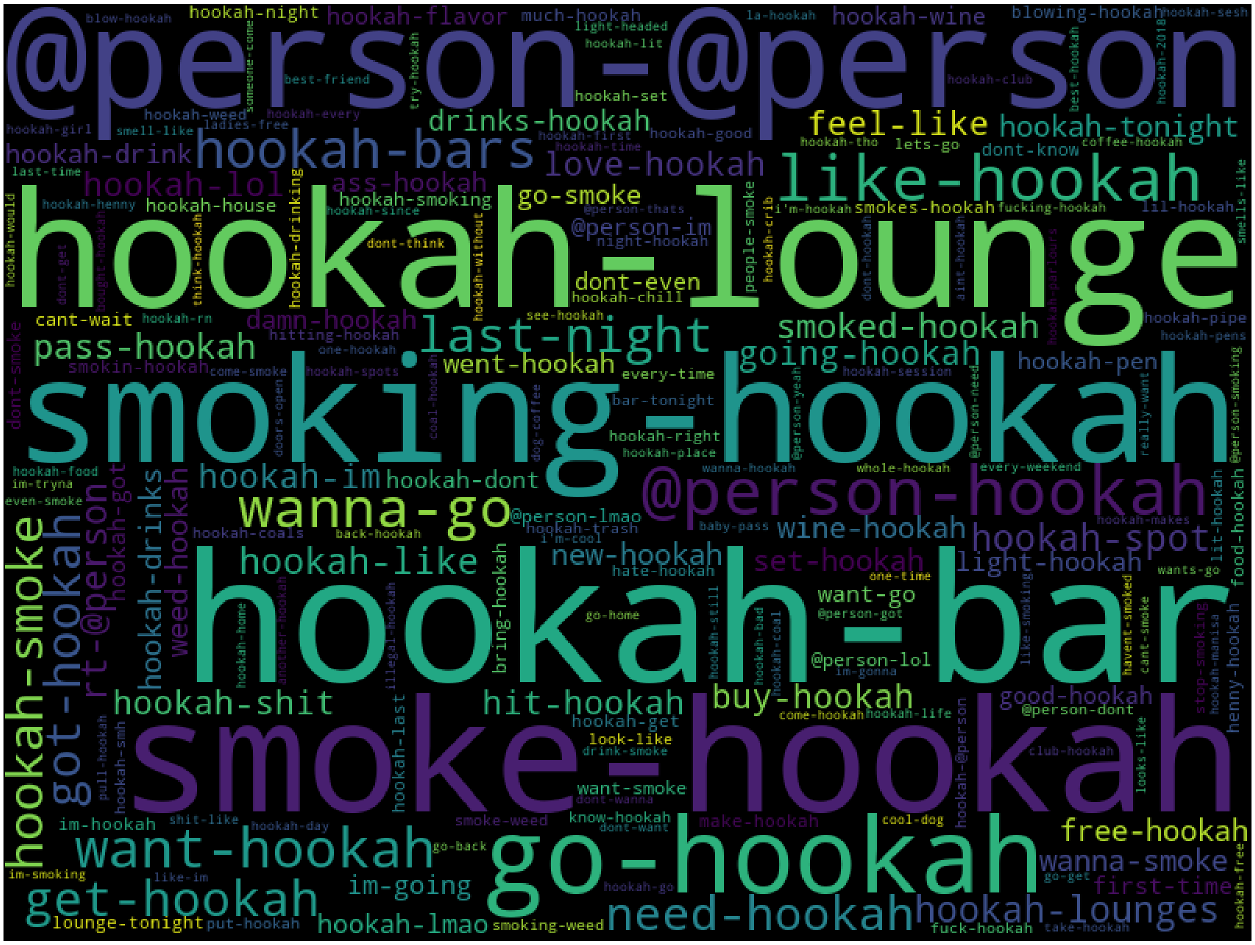

February 2018

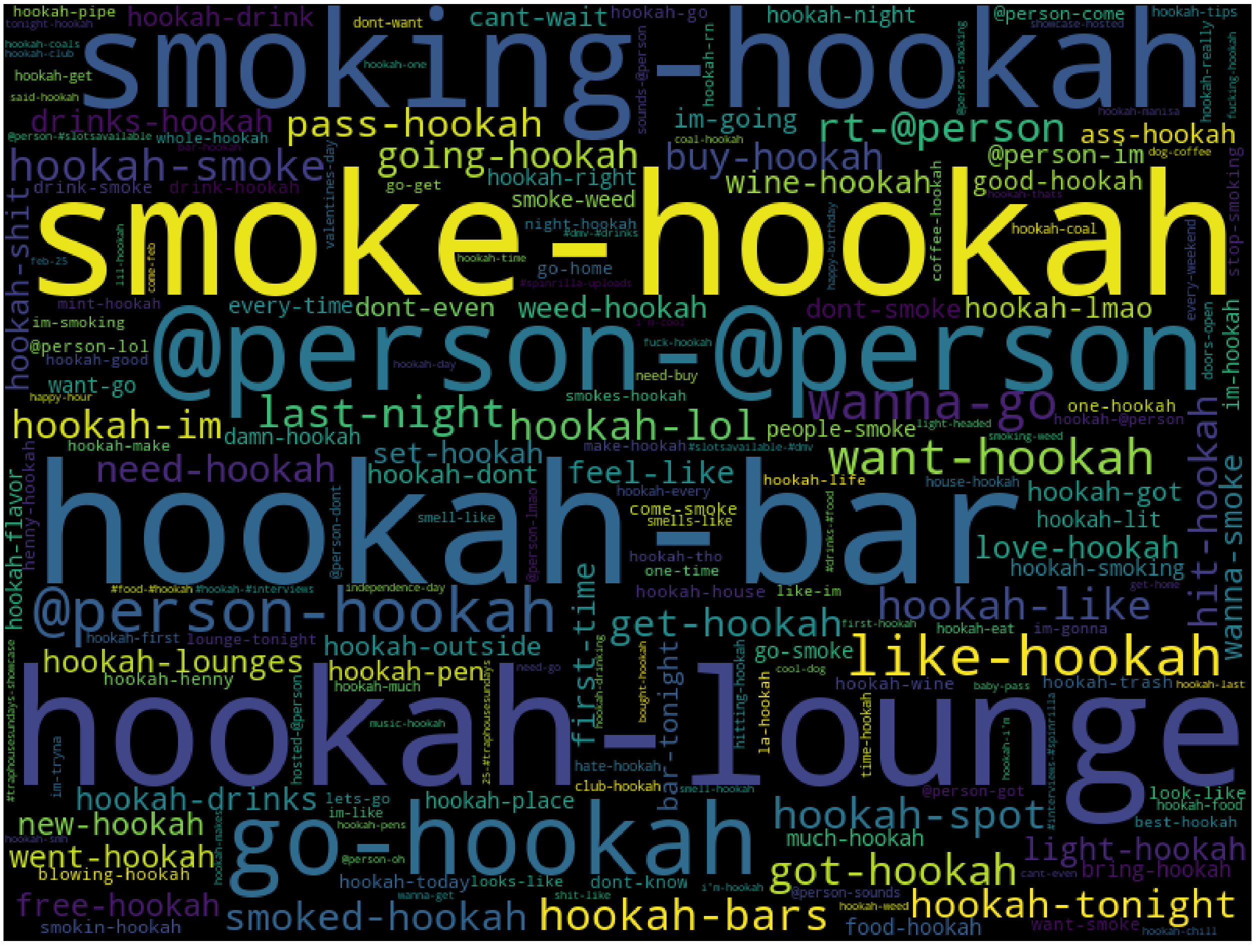

March 2018

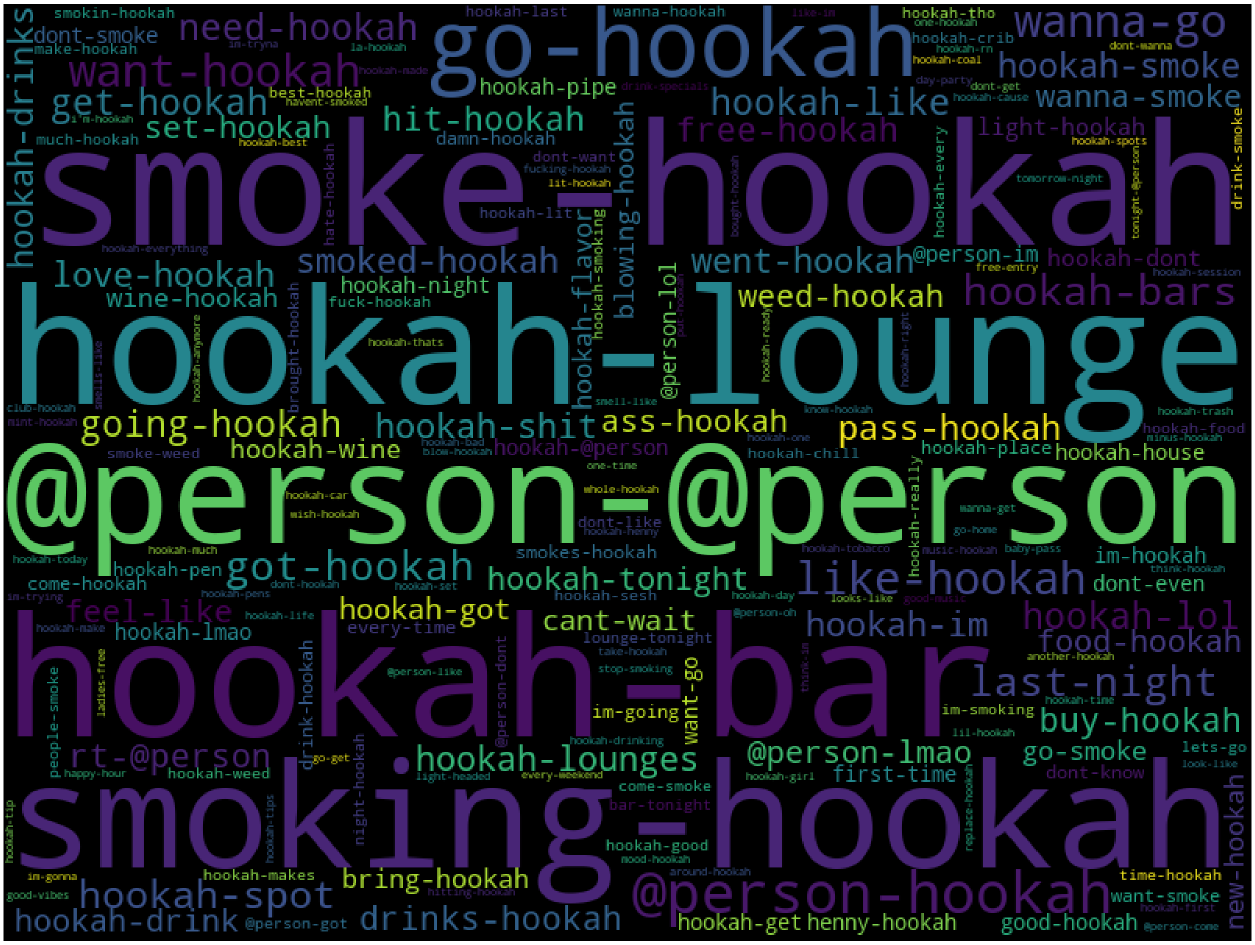
smoke-hookah
hookah-lounge
@person-@person
hookah-bar
smoking-hookah
go-hookah
want-hookah
need-hookah
wanna-go
hookah-smoke
wanna-smoke
hookah-like
get-hookah
set-hookah
hit-hookah
free-hookah
light-hookah
hookah-drinks
love-hookah
smoked-hookah
went-hookah
hookah-bars
wine-hookah
weed-hookah
hookah-night
hookah-flavor
blowing-hookah
@person-lol
@person-im
hookah-dont
going-hookah
hookah-shit
ass-hookah
pass-hookah
hookah-wine
hookah-@person
hookah-place
hookah-house
hookah-food
got-hookah
hookah-tonight
like-hookah
im-hookah
dont-even
hookah-pen
come-hookah
smokes-hookah
hookah-got
cant-wait
hookah-im
hookah-lol
food-hookah
feel-like
hookah-lmao
last-night
buy-hookah
rt-@person
hookah-lounges
@person-lmao
go-smoke
first-time
dont-know
hookah-weed
im-going
want-go
drink-hookah
night-hookah
@person-hookah
hookah-spot
bring-hookah
hookah-drink
drinks-hookah
hookah-get
henny-hookah
good-hookah
want-smoke
time-hookah
new-hookah
hookah-good
hookah-makes
hookah-pipe
damn-hookah
dont-smoke
smokin-hookah
make-hookah
much-hookah
best-hookah
hookah-last
wanna-hookah
hookah-tho
hookah-crib
drink-smoke
hookah-every
brought-hookah
hate-hookah
fuck-hookah
smoke-weed
hookah-chill
dont-like
come-smoke
bar-tonight
lounge-tonight
every-time
im-smoking
lets-go
hookah-really
people-smoke
hookah-sesh
hookah-trash
